# Realizing the potential of artificial intelligence in healthcare: Learning from intervention, innovation, implementation and improvement sciences

**DOI:** 10.3389/frhs.2022.961475

**Published:** 2022-09-15

**Authors:** Per Nilsen, Julie Reed, Monika Nair, Carl Savage, Carl Macrae, James Barlow, Petra Svedberg, Ingrid Larsson, Lina Lundgren, Jens Nygren

**Affiliations:** ^1^School of Health and Welfare, Halmstad University, Halmstad, Sweden; ^2^Department of Health, Medicine and Caring Sciences, Linköping University, Linköping, Sweden; ^3^Department of Learning, Informatics, Management and Ethics, Medical Management Centre, Karolinska Institutet, Stockholm, Sweden; ^4^Centre for Health Innovation, Leadership and Learning, Nottingham University Business School, Nottingham, United Kingdom; ^5^Centre for Health Economics and Policy Innovation, Imperial College Business School, London, United Kingdom

**Keywords:** artificial intelligence, intervention, innovation, implementation, improvement

## Abstract

**Introduction:**

Artificial intelligence (AI) is widely seen as critical for tackling fundamental challenges faced by health systems. However, research is scant on the factors that influence the implementation and routine use of AI in healthcare, how AI may interact with the context in which it is implemented, and how it can contribute to wider health system goals. We propose that AI development can benefit from knowledge generated in four scientific fields: intervention, innovation, implementation and improvement sciences.

**Aim:**

The aim of this paper is to briefly describe the four fields and to identify potentially relevant knowledge from these fields that can be utilized for understanding and/or facilitating the use of AI in healthcare. The paper is based on the authors' experience and expertise in intervention, innovation, implementation, and improvement sciences, and a selective literature review.

**Utilizing knowledge from the four fields:**

The four fields have generated a wealth of often-overlapping knowledge, some of which we propose has considerable relevance for understanding and/or facilitating the use of AI in healthcare.

**Conclusion:**

Knowledge derived from intervention, innovation, implementation, and improvement sciences provides a head start for research on the use of AI in healthcare, yet the extent to which this knowledge can be repurposed in AI studies cannot be taken for granted. Thus, when taking advantage of insights in the four fields, it is important to also be explorative and use inductive research approaches to generate knowledge that can contribute toward realizing the potential of AI in healthcare.

## Introduction

Artificial intelligence (AI) is widely seen as critical for tackling fundamental challenges faced by health systems, including increasing demand and higher costs, workforce pressures and limited resources, and the need to deliver high-quality patient outcomes and experiences (1–3). AI is an umbrella term that refers to the application of machine learning and other cognitive technologies to perform tasks or reasoning processes that are usually associated with human intelligence (4). In recent years, there have been promising advances in AI-based applications in healthcare, drawing on enhanced computing power and vast amounts of digital data becoming available for analysis (5).

AI includes techniques to improve the ability to detect and predict various conditions, with implications for screening, assessment and clinical decision-making (6). AI can be used to improve health by identifying individual treatments or treatment elements most likely to provide benefit for a particular person, thus promoting personalized healthcare (7). AI can also be incorporated into digital tools, such as smartphone apps and health wearables, to create novel interventions, thus reducing reliance on care processes and actions taken by healthcare professionals (8). Nevertheless, healthcare has been slow to make use of AI compared with other societal sectors (9–11).

AI has predominantly been viewed through a rather narrow technology-centric lens, with research focusing on the design of the technology and its interaction with the immediate users (12–14). However, some of the major challenges in AI are faced in the “last mile” of the AI research and development (R&D) process, i.e., the implementation and routine use of AI-based applications in clinical settings (15, 16). Research is scant on the factors that influence the implementation and routine use of AI in healthcare, how AI may interact with the context in which it is implemented, and how it can contribute to wider health system goals (17).

Deploying and using AI in healthcare could benefit from the extensive knowledge about health technologies and new practices that has been generated in four established fields of science: intervention, innovation, implementation, and improvement sciences. First, AI-based applications, e.g., AI incorporated into digital tools, can function as an intervention with the aim of improving patient health outcomes by means of better detection, prevention, treatment, and monitoring of health and ill-health. Second, AI-based applications in healthcare represent an innovation when adopted and used in clinical practice because they introduce novel processes and practices. Third, implementation of innovations and new interventions in healthcare typically require supportive strategies because there are often barriers to their use in the form of healthcare professionals' existing habits, routines, work processes and professional cultures that tend to be difficult to change. Fourth, how AI-based applications will contribute to the improvement goals of the broader health system and fit with overall care delivery practices and processes need to be considered.

We propose that AI development can benefit from knowledge generated in these four scientific fields to realize the potential of AI and accelerate its use in healthcare. Thus, the aim of this paper is to briefly describe intervention, innovation, implementation, and improvement sciences, and to identify potentially relevant knowledge from the four fields that can be utilized for understanding and/or facilitating the use of AI in healthcare. The paper is based on the authors' experience and expertise in the four fields and a selective literature review.

## Utilizing knowledge from the four fields

### Intervention science

Intervention science is the study of purposive efforts (i.e., interventions) to change the natural order of things or a foreseeable sequence of events. Intervene literally means “to come between,” from Latin *inter* (“between”) and *venire* (“to come”) (18). Health intervention science starts with the premise that each intervention, such as an AI-based application, is seeking to address a specific health issue. The aim is to establish the extent to which or whether a desirable outcome is achieved. The development of evidence for various treatments, therapies, procedures, and actions to improve health-related outcomes is an important goal for intervention studies in healthcare contexts (19). It is also important to assess the appropriateness, acceptability, and feasibility of interventions because these characteristics influence the degree to which it is possible to use these interventions in routine practice (20–22).

An important goal of health intervention research is to contribute to a more evidence-based healthcare practice, which is relevant to the study of AI-based applications in healthcare, i.e., evidence for their effectiveness in having an impact on various health-related problems. However, interventions also need to account for risks and other aspects beyond the intended health-related goals, thus balancing the need for evidence with other meaningful and value-creating outcomes (19), e.g., person-centered care and shared decision-making.

Interventions need to address health issues in a way that makes it possible to put them into routine healthcare use if proven effective (19). Intervention science involves both efficacy studies (can it work?) conducted under ideal controlled conditions to establish internal validity, and effectiveness studies (does it work in practice?) conducted under more realistic real-world conditions, with more emphasis on external validity (while still retaining internal validity) (22). External validity is threatened if interventions are delivered in resource-intensive pilot studies that are difficult to scale up (23, 24) or if the interventions are provided by unrepresentative healthcare professionals (e.g., perhaps having very experienced staff or receiving extensive training in delivering the intervention) and if the patients are atypical of routine practice (e.g., due to restrictive exclusion criteria) (25). Like other interventions, AI-supported interventions should be investigated in effectiveness research after efficacy has been established.

### Innovation science

Innovation science is the study of how problem-solving ideas, processes, products, and services are developed, diffused, and adopted by various stakeholders, ranging from individuals through organizations to whole systems. The aim is to understand how innovations can contribute to increased value, productivity, profitability, competitiveness and other desirable goals (26). Innovation can be conceptualized in several dimensions: stages of the innovation process (e.g., R&D, efficacy and market validation, regulatory approval, adoption, diffusion), level of analysis (e.g., individuals, organizations, and systems), and type (e.g., product versus process innovations and incremental versus radical innovations). Innovation is new knowledge incorporated into ideas, processes, products and services. However, an innovation does not need to be objectively new; it is sufficient that it is perceived as new in a specific context by individual adopters (e.g., healthcare professionals) or adopting units (e.g., a hospital) (27).

An important strand of innovation research focuses on adoption and diffusion, exploring the factors that influence the way in which innovations are brought into use. Factors may be related to the attributes of innovations themselves or to the wider context for adoption (27). Typically, the innovation literature argues that innovations are more likely to be adopted if they are perceived to have low complexity but high compatibility with existing values, beliefs, past experiences, and needs of potential users (28). Adoption has also been linked with the relative advantage of the innovation over existing processes or practices (e.g., the innovation is more economical), trialability (the degree to which an innovation may be experimented with on a limited basis), and observability (the degree to which the results of an innovation are visible) (27).

Adoption of certain innovations may often be more challenging than the R&D process (29). This is particularly the case in healthcare, as shown in an increasing body of innovation research accounting for the specificities of the healthcare sector (30). This research emphasizes the importance of a thorough understanding of contextual conditions, such as the way key stakeholders, including healthcare professionals, patients, and managers, influence adoption decisions (31, 32). This is relevant to the use of AI in healthcare because the system within which AI innovations are being introduced is complex and highly regulated.

Other areas within innovation science center on service innovations (33) and business model innovations (34). AI systems built into physical products, such as health wearables or which enhance the processes involved in providing a healthcare service, have the potential to alter or disrupt existing service and business models. A strand of innovation research addresses issues such as user involvement and how leadership, strategy, and management influence success (35), and how organizations innovate in their value propositions and their underlying operating models (36).

### Implementation science

Implementation science as a field emerged in response to the challenges of putting evidence-based interventions and innovations into routine practice. The focus is on studying barriers and facilitators to implementing evidence-based practices (e.g., interventions, programs, and services) and evaluating the effectiveness of strategies to support implementation of such practices. The aim is to reduce the knowing-doing gap in healthcare and other sectors of society, thus improving patient and population outcomes (37). The word “implementation” is derived from the Latin *implere*, meaning to fulfill or carry into effect, which provides a basis for a broad definition of implementation science as the study of the uptake of innovations and interventions into everyday use in healthcare and other settings (38).

Implementation science has established the importance of considering implementation from the outset and planning to create conditions conducive for implementation (39). Process models have been developed in the field to describe important activities, including various supportive strategies (i.e., interventions directed at healthcare professionals and/or the organization), to be undertaken during the implementation process (40). Preparing for implementation is likely to be just as important when various AI-based applications are considered for use in healthcare settings.

Implementation science has largely focused on implementation of various evidence-based practices, typically different forms of health interventions with support for their efficacy and effectiveness established in empirical research (41). However, a key lesson in implementation science is that evidence is not sufficient to ascertain real-world use. Rather, there is a need for implementation strategies to support implementation. Taxonomies of strategies and their effectiveness to influence implementation outcomes have been assembled in the field, but the results tend to be highly context-dependent (42).

Frameworks have been developed in the field to identify and structure determinants (i.e., barriers and facilitators) of implementation success. The field has borrowed the concept of innovation attributes from innovation science (40), but has added healthcare-specific attributes such as perceptions of evidence strength and quality and source of the intervention (e.g., developed externally or internally) (43). Knowledge about the determinants provides input for selecting the most appropriate strategies to overcome barriers and/or harness facilitators (44).

Outcomes in implementation science include adoption (i.e., decision or action to use an intervention), fidelity (degree to which an intervention was implemented as intended), sustainability, and cost. There is some overlap with intervention science because the acceptability, appropriateness, and feasibility of an intervention are considered proximal outcomes that predict adoption and other more distal outcomes such as sustainability (20).

### Improvement science

Improvement science refers to the study and practice of achieving improvements in complex systems, such as healthcare. The field has a similar aim to implementation science, understanding the gap between ideal and actual care and bridging this gap by improving healthcare quality within local practices, processes, and culture. However, improvement science extends beyond implementation of evidence-based practices to consider the quality of wider system performance (e.g., safety, timeliness, effectiveness, efficiency, equality, patient-centeredness) (45). The field has grown out of the wider quality improvement movement, which entered healthcare in the late 1980s (46, 47).

Improvement science provides a holistic approach to health system improvement, of which an AI system or application may be just one complex intervention within a wider multifaceted attempt to improve a system. Improvement approaches advocate a process that begins with exploring the problems and opportunities within a specific setting, which could inform what AI-based applications might be relevant and what features would be required to make them useful and effective. The next step involves identifying and testing potential interventions (e.g., AI-based applications) alongside other interventions that may be required to improve quality using a process of informed iterative development until an improvement goal is achieved. This is followed by embedding a process of continual improvement to sustain initial improvements and respond to emerging problems and opportunities (48).

The importance of understanding local context and its influence on outcomes is a cornerstone of improvement science (49). Understanding, influencing, and adapting to local context, e.g., practices, processes and cultures, is critical to improvement. Thus, the people within each local system, e.g., healthcare professionals, managers, and patients, are seen as important stakeholders to achieve improvement. Improvement approaches aim to enable them to take a structured, systematic approach to navigating the change process (48).

Improvement science offers both a way of thinking and a methodological toolkit that might be useful to understand, adapt, and apply AI-based applications in various healthcare contexts. A range of approaches and tools are used to understand problems and to inform and evaluate solution designs and efforts to facilitate their application in practice, e.g., Plan-Do-Study-Act cycles, Six-Sigma, Root Cause Analysis, Process Mapping, and simulation (50–53). These approaches and tools can be used to enable people to understand their local contexts and define important outcomes (e.g., improved patient safety, patient outcomes, reduced costs) and to intervene in the health system to improve overall performance (54).

## Discussion

The four fields of intervention, innovation, implementation, and improvement sciences have generated a wealth of often-overlapping knowledge, some of which we propose have considerable relevance for understanding and/or facilitating the use of AI in healthcare (Table 1). Thus far, AI research has largely focused on engineering, computer science and programming (12–14). However, this research does not ascertain the adoption and subsequent use of AI in everyday healthcare. Implementation and use of new interventions and innovations in healthcare often face considerable organizational inertia and skepticism or even resistance from leaders, physicians and nurses (38, 39, 43). Thus, the four fields have an important role in the future of AI in healthcare because they generate knowledge based on studying interventions and innovations in the real-world context in which they are introduced and used. Research is needed to investigate the effectiveness of AI-based applications when efficacy studies have established that they work. Real-world evidence of AI-based applications is important because it provides a more comprehensive understanding of how new practices will work under realistic healthcare conditions and makes it possible to establish whether they contribute to the intended improvements in care quality and other desirable outcomes.

**Table 1:** The four scientific fields' aims, research characteristics, and relevance for AI in healthcare.

**Scientific field**	**Aims of the research**	**Characteristics of the research**	**Relevance in the AI invention, planning and realization stages**	**Examples of research question of relevance for the understanding and/or facilitation of AI use in healthcare**
Intervention science	To establish the extent to which or whether a desirable clinical, health-related, cost-effectiveness or other outcomes is achieved by an intervention	Studies to evaluate the efficacy and effectiveness of interventions	Applicable both to the planning stage (e.g., efficacy studies) and the realization stage (e.g., effectiveness studies)	• What is the efficacy of AI-based applications under more ideal conditions? • What is the effectiveness of AI-based applications under routine healthcare conditions? • To what extent do AI-based applications contribute to other outcomes such as a person-centered care and shared decision-making?
Innovation science	To understand how innovation can contribute to increased value, productivity, profitability, competitiveness and other desirable goals	Studies to investigate how problem-solving ideas, processes, products and services are developed, diffused, and adopted by various stakeholders	Applicable both to the planning stage (e.g. studies of factors influencing adoption decisions) and the realization stage (e.g. studies of the influence of contextual conditions and stakeholders)	• What AI-related factors influence adoption decisions concerning AI in healthcare? • What contextual conditions and stakeholders influence adoption decisions concerning AI in healthcare? • How can AI change existing healthcare service and business models?
Implementation science	To understand the knowing-doing gap and facilitate the reduction of the gap	Studies to identify barriers and facilitators to implement evidence-based practices and to evaluate the effectiveness of strategies to support implementation of such practices	Primarily applicable in the realization stage	• How can it be ascertained that AI in healthcare is planned from the outset to create conditions conducive to implementation? • What are the barriers and facilitators to implementing AI in healthcare? • What strategies can be used to support the implementation of AI in healthcare and how effective are these strategies?
Improvement science	To understand the gap between ideal and actual care and bridge this gap by improving healthcare quality within local practices, processes, and culture	Studies to investigate the quality of complex systems, e.g. in terms of safety, timeliness, effectiveness, efficiency, equality and patient-centeredness and other goals	Primarily applicable in the realization stage	• What are the problems and opportunities to achieving a process of continual improvement with regard to AI in healthcare? • How does AI contribute to system improvement in terms of safety, timeliness, effectiveness, efficiency, equality, patient-centeredness and other goals? • How can the context and stakeholders in the local system in which AI is used contribute to healthcare system improvement?

Knowledge from the four fields is primarily relevant to the latter stages of the innovation process, which is often described in terms of three overarching stages, from invention to development and realization. The development stage typically encompasses various forms of prototyping, testing and efficacy studies conducted under more controlled conditions (55, 56). Knowledge generated in intervention and innovation sciences is important for this stage. The realization stage can be distinguished into introduction, operation and refinement (55, 56). Implementation science knowledge is particularly useful when introducing AI, while improvement science knowledge is useful for improving operation and refining AI systems and AI-based applications. However, the stages are interdependent and knowledge from the four fields largely overlaps. For example, implementation science involves studies of various implementability characteristics (e.g., feasibility), which has more to do with planning than with realization. Conversely, intervention science involves studies of effectiveness conducted under real-world conditions, which is more relevant in the realization stage than the planning stage.

Intervention science underscores the relevance of taking a broad perspective on intervention goals. Hence, the effectiveness of AI-based applications should not be in conflict with other important goals, such as patient involvement in their care or trusting communication between the healthcare professional and patient. Implementation science points to the importance of understanding the use of interventions in routine practice, e.g., how AI-derived information is interpreted and used by healthcare professionals. Similarly, improvement science emphasizes the relevance of studying AI-based applications in a larger context of how they can contribute to wider health system goals. Intervention and implementation sciences highlight the importance of the acceptability, appropriateness, and feasibility of interventions to enable their routine use in healthcare. These outcomes and others described in implementation science such as fidelity, sustainability, and costs are important to investigate with regard to AI-based applications.

The four fields address the influence of context on interventions and innovations to varying degrees. The use of AI in healthcare represents a sociotechnical system that requires contextual understanding of the interrelations of social and technical aspects of the organization as a whole (57). Applications of AI in healthcare can circumvent traditional workflow and care delivery pathways (58). Improvement science is particularly concerned with how practices are integrated within the local context and how they might interact with and/or disrupt existing work practices and processes.

Innovation, implementation, and improvement sciences further emphasize the role of stakeholders and other contextual influences, which is relevant because the decisions involved in the introduction and use of some AI-based applications in healthcare may involve many stakeholders and have widespread or disruptive impact across the health system. On the other hand, there may also be AI-based applications that are simple, uncontroversial, involve few stakeholders, and have limited impact outside the immediate processes in which the applications are used. Implementation determinants tend to be multi-factorial and multi-level (43), which suggests the relevance of using strategies to support AI implementation that address multiple determinants at different levels of the health system.

The development and deployment of AI represents a complex sociotechnical process that spans work associated with data acquisition, algorithm selection and development, organizational design, professional education, and associated governance and regulatory activities (59). This sociotechnical complexity complicates the study and practice of AI in healthcare. New studies drawing on empirical and theoretical knowledge from the four fields will need to consider whether and how to distinguish between the general requirements of any AI system and the specific requirements for any particular type of AI-based application. AI is not one single type of technology but rather encompasses many technologies covering a range of practical applications (10, 60). For example, AI-based applications may, or may not, be perceived as complex and/or compatible (i.e., two key innovation attributes), depending on what the application is and what context it is used in; e.g., breast cancer or cataract screening vs. algorithms to help decision-making about mental health therapies. This may limit the usefulness of generalizable findings, and it is likely to also be important to investigate determinants in relation to specific AI-based applications.

There might be a need to develop new theoretical approaches or augment and re-contextualize existing ones from the four fields. Frameworks have been developed for implementation of healthcare technologies, e.g., the Non-adoption, Abandonment, Scale-up, Spread, Sustainability (NASSS) framework (61), but it has been shown that existing frameworks do not consider all of the specific issues relevant to AI systems and AI-based applications (17). Development of bespoke AI frameworks would benefit from interdisciplinary work by researchers in the four fields and collaboration with healthcare professionals, AI developers, patients, policymakers, and other stakeholders.

This paper has addressed knowledge from intervention, innovation, implementation and improvement sciences of potential importance for understanding and/or facilitating AI use in healthcare. However, the scope of the paper has obvious limitations and it does not address everything that might have relevance when introducing and using AI in healthcare, e.g., issues related to data integrity, transparency, governance and reimbursement. AI has a wide range of applications beyond healthcare and health, including in domains such as agriculture, engineering, commerce, marketing, finance, gaming, education, navigation, and transportation (62). The broad use of AI in many different sectors and areas of life requires multidisciplinary knowledge from many fields other than those covered in this paper to address issues such as ethics, fairness, equity, accountability, protection of privacy and respect, transparency, trustworthiness and auditing (63–65).

In conclusion, the four fields of intervention, innovation, implementation, and improvement sciences have generated many insights of potential relevance for understanding and/or facilitating the use of AI in healthcare. Knowledge derived from these fields provides a head start for research on the use of AI in healthcare, but the extent to which this knowledge can be repurposed in AI studies cannot be taken for granted. Thus, when taking advantage of insights in the four fields, it is important to also be explorative and use inductive research approaches to generate knowledge that can contribute toward realizing the potential of AI in healthcare.

## Data availability statement

The original contributions presented in the study are included in the article/supplementary material, further inquiries can be directed to the corresponding author/s.

## Author contributions

PN conceptualized the study, but all aspects were discussed with the other authors and he drafted the first version of the manuscript with input from all authors. All authors discussed further drafts, revised the manuscript, and approved the final manuscript.

## Funding

The funders for this study are the Swedish Government Innovation Agency Vinnova (grant 2019-04526) and the Knowledge Foundation (grant 20200208 01H).

## Conflict of interest

The authors declare that the research was conducted in the absence of any commercial or financial relationships that could be construed as a potential conflict of interest.

## Publisher's note

All claims expressed in this article are solely those of the authors and do not necessarily represent those of their affiliated organizations, or those of the publisher, the editors and the reviewers. Any product that may be evaluated in this article, or claim that may be made by its manufacturer, is not guaranteed or endorsed by the publisher.
